# Deterministic Effects Propagation Networks for reconstructing protein signaling networks from multiple interventions

**DOI:** 10.1186/1471-2105-10-322

**Published:** 2009-10-08

**Authors:** Holger Fröhlich, Özgür Sahin, Dorit Arlt, Christian Bender, Tim Beißbarth

**Affiliations:** 1 German Cancer Research Center, Molecular Genome Analysis, Im Neuenheimer Feld 280, 69120 Heidelberg, Germany; 2 University Medicine Gottingen, Medical Statistics, 37099 Göttingen, Germany

## Abstract

**Background:**

Modern gene perturbation techniques, like RNA interference (RNAi), enable us to study effects of targeted interventions in cells efficiently. In combination with mRNA or protein expression data this allows to gain insights into the behavior of complex biological systems.

**Results:**

In this paper, we propose Deterministic Effects Propagation Networks (DEPNs) as a special Bayesian Network approach to reverse engineer signaling networks from a combination of protein expression and perturbation data. DEPNs allow to reconstruct protein networks based on combinatorial intervention effects, which are monitored via changes of the protein expression or activation over one or a few time points. Our implementation of DEPNs allows for latent network nodes (i.e. proteins without measurements) and has a built in mechanism to impute missing data. The robustness of our approach was tested on simulated data. We applied DEPNs to reconstruct the *ERBB *signaling network in *de novo *trastuzumab resistant human breast cancer cells, where protein expression was monitored on Reverse Phase Protein Arrays (RPPAs) after knockdown of network proteins using RNAi.

**Conclusion:**

DEPNs offer a robust, efficient and simple approach to infer protein signaling networks from multiple interventions. The method as well as the data have been made part of the latest version of the R package "nem" available as a supplement to this paper and via the Bioconductor repository.

## Background

Reverse engineering of biological networks is a key for the understanding of biological systems. The exact knowledge of interdependencies between proteins in the living cell is crucial for the identification of drug targets for various diseases. However, due to the complexity of the system a complete picture with detailed knowledge of the behavior about the individual proteins is still in the far future. Nonetheless, the advent of gene perturbation techniques, like RNA interference (RNAi) [1], has enabled the possibility to study cellular systems systematically under varying conditions, hence opening new perspectives for network reconstruction methods.

A number of approaches have been proposed in the literature for estimating networks from perturbation effects. Many of these approaches aim at reconstructing a network from directly observable effects. For example, Rung et al. [2] builds a directed disruption graph by drawing an edge (*i*, *j*), if gene *i *results in a significant expression change at gene *j*. Wagner [3] uses such disruption networks as a starting point for a further graph-theoretic method, which removes indirect effects [4], hence making the network more parsimonious. Tresch at el. [5] extend this approach by additionally making use of *p-*values and fold-change directions to make the network more consistent with the observed biological effects.

Also Bayesian Networks have been used to model the statistical dependency between perturbation experiments [6,7]. For this purpose Pearl [8] proposes an idealized model of interventions. He assumes that once a network node is manipulated, the influence of all parent nodes is eliminated and the local probability distribution of the node becomes a point mass at the target state. Besides for Bayesian Networks, ideal interventions have also been applied for factor graphs [9] and dependency networks [10].

Epistasis analysis offers a possibility for learning from indirect downstream effects. For example, Driessche et al. [11] use expression profiles from single and double knockdowns to partly reconstruct a developmental pathway in *D. discoideum *via a simple cluster analysis.

Also fully quantitative models using differential equation systems have been suggested. For example, Nelander et al. [12] propose a model for predicting combinatorial drug treatment effects in cancer cells.

Recently, *Nested Effects Models *(NEMs) [13-21] have been proposed as a method, which is specifically designed to learn the signaling flow between perturbed genes from indirect, high-dimensional effects, typically monitored via DNA microarrays. NEMs use a probabilistic framework to compare a given network hypothesis with the observed nested structure of downstream effects. Perturbing one gene may have an influence on a number of downstream genes, while perturbing others affects a subset of those. Moreover, several of these subsets could be disjoint, i.e. the knockdown of gene *i *shows effects, which mostly do not overlap with the effects seen at the knockdown of gene *j*. NEMs have been applied successfully to data on immune response in *Drosophila melanogaster *[13], to the transcription factor network in *Saccharomices cerevisiae *[14], to the ER-*α *pathway in human breast cancer cells [16,17], and to the synthetic lethality interactions network in *Saccharomicies cerevisiae *[18].

The work presented in this paper is designed for a different scenario: We would like to reverse engineer a protein signaling network based on experimentally measured effects on protein expression and activation level after multiple interventions. These interventions may also be combinatorial [22], i.e. there is more than one knock-down at a time. Importantly, the set of all perturbations should cover a fraction as large as possible of the network proteins.

Effects of all interventions on the network proteins are quantified directly on protein expression and activation level, for instance via Reverse Phase Protein Arrays (RPPAs) [23]. Here, we propose a probabilistic approach called Deterministic Effects Propagation Networks (DEPNs), which can estimate the most likely signaling network based on these data. DEPNs are a special case of Bayesian Networks, which employ a mixture of purely deterministic and Gaussian variables. While DEPNs and NEMs have a similar effects propagation scheme, DEPNs differ from NEMs with respect to the following aspects:

• In NEMs each node corresponds to one perturbation experiment. In DEPNs each node corresponds to one single protein, potentially influenced by one or several perturbations.

• NEMs only work, if the number of perturbations is much smaller than the number of monitored downstream effects. In DEPNs the opposite is true: DEPNs assume that at least each network node has been perturbed once. More perturbations are beneficial.

• In NEMs indirect, high dimensional downstream intervention effects are monitored. In DEPNs intervention effects are monitored on all other network proteins (i.e. they are typically of low dimension).

• DEPNs offer the possibility to include combinatorial interventions in addition to single interventions in a natural way.

• DEPNs can use latent network nodes (proteins without effects measurements, which have been perturbed). This has not been introduced for NEMs so far.

• DEPNs offer a mechanism for missing value imputation. This has not been introduced for NEMs so far as well.

In contrast to other Bayesian Networks, DEPNs allow for directed graph structures with loops, because they employ a deterministic effects propagation scheme. Our approach was first validated using an extensive simulation study. Afterwards it was employed to reverse engineer the *ERBB *receptor regulated G1/S transition network in HCC1954 human breast cancer cells, which show *de novo *resistance against trastuzumab (a monoclonal antibody targeting the *ERBB2 *receptor in *ERBB2 *overexpressing cells [24]). In this dataset [25] knock-downs of 16 proteins utilizing RNAi were performed and effects were measured via RPPAs.

## Results and Discussion

### Deterministic Effects Propagation Networks

Our basic assumption is that we measure an unknown signaling network of proteins by performing multiple interventions on these proteins. These perturbations may affect single network proteins at a time or may also be combinatorial, i.e. they affect several network proteins at one time. For each network protein in the network we monitor the effect of all interventions carried out on proteins in the network. Note, that each specific intervention not only influences direct targets, but may also cause effects on downstream network proteins. We explicitly assume that in one experiment all network proteins are unperturbed.

We employ special instances of Bayesian Networks, which we call Deterministic Effects Propagation Networks (DEPNs), to infer the most likely protein network given measurements from multiple interventions under different experimental conditions (e.g. stimulated/unstimulated) or time points. Our implementation of DEPNs can deal with latent network nodes (proteins without measurements) and with missing data. The details of our method are described in the Section Material and Methods. Briefly, the idea is that we have an unknown network graph, where each node can have two states: perturbed and unperturbed. In principle, this could also be extended to three states (activated/inhibited/unperturbed), but in this paper we only deal with the simpler binary case. Furthermore, attached to each node we have experimental measurements, which for each time point are assumed to come from two Gaussian distributions, one for the perturbed and one for the unperturbed case. The parameters of these distributions are determined in the following way:

1. For each perturbation experiment we compute the expected downstream effects. This is done by assuming node *i *to be perturbed whenever *i *itself or any of its parents are perturbed, i.e. perturbation effects are propagated in a deterministic fashion from parents to children. This implies that the network structures learned by DEPNs are always transitively closed graphs: If there is a path from *a *to *b *then there is also an edge *a *→ *b*. It should to mentioned that the perturbations state of a protein can never depend on itself through a loop in our model, i.e. when the state of a variable is obtained it is not updated further. However, this behavior could be changed in principle.

2. Since we have several perturbation experiments and also one control experiment, we can now determine for each protein measurement, whether the protein was perturbed or not.

3. With this information the data for each protein gets divided into two distributions: one for the perturbed and one for the unperturbed case. The parameters of these distributions, which are supposed to be Gaussian, are estimated either in a maximum likelihood or in a Bayesian fashion. The details are described in the Material and Methods Section.

Given the parameters we can then calculate the probability of observing the data for all proteins under a given network hypothesis. An edge *a *→ *b *in this network denotes an influence of *a *on *b*. This may be interpreted as the direction of the signal flow. Our model does not distinguish between signaling via a transcriptional regulation or via a protein phosphorylation. The type of the edge is dependent on the type of antibody used (i.e. measurement of protein expression levels or of phosphorylation/activation levels).

**Figure 1:**
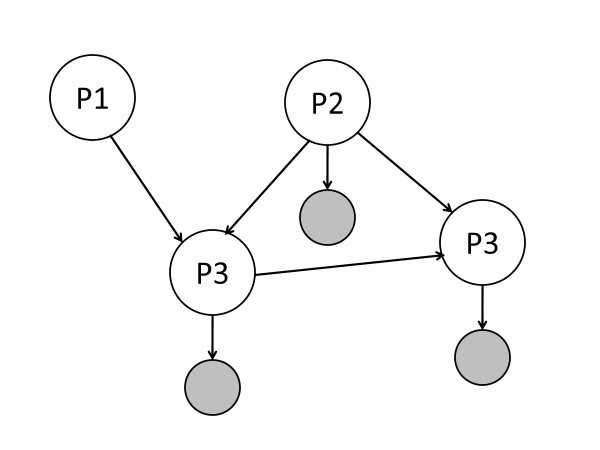
**Schematic view on DEPNs as a special type of Bayesian Networks: White nodes (proteins) have a deterministic state, whereas grey nodes (measurements) are Gaussian**. Combinatorial intervention effects are propagated in a deterministic way between proteins. Measurements are only affected indirectly and are conditionally independent from each other. Protein P1 has no associated measurements and is therefore a latent node.

Figure 1 shows a graphical representation of DEPNs as a special type of a Bayesian Network. Protein nodes (white) can be either perturbed or unperturbed by the application of a specific intervention, i.e. they are deterministic. In contrast, measurement nodes (grey) attached to proteins are Gaussian.

If the number of nodes in the network is considerably small (e.g. < 5), we can enumerate all possible network hypothesis and take the one with the highest likelihood. However, this exhaustive search will be impossible as soon as the network gets larger. For instance, for 10 nodes there are already 10^27 ^network hypothesis to test. Therefore, here we restrict ourselves to a greedy hill climbing approach, in which we begin with an initial network (usually the empty network, if not stated otherwise) and then successively add the edge (and all transitively implied edges) increasing the likelihood most. All possible alternatives are checked. We do not use any arbitrary starting point for the hill climber, because DEPNs operate in the space of transitively closed graphs only. Therefore, no backtracking/edge removal step can be implemented in an obvious way. Hence, initial networks should be chosen in a sensible way.

### Simulation Studies

#### Graph Sampling

We tested the performance of our approach in a simulation study on artificial data, which were created as follows: Subgraphs with *n *= 6 and *n *= 10 nodes were cut out randomly from randomly selected KEGG [26] signal transduction pathways. Only gene-gene interactions were considered as edges in the graph. Please note that our sampled subgraphs are not random graphs, but randomly selected existing subgraphs of KEGG pathways. For *n *= 6 96.75% of the sampled subgraphs were acyclic. For *n *= 10 these were 85.5%.

#### Data Simulation

Single knock-downs of all individual network proteins as well as one experiment without interventions were simulated as follows: For each simulated experiment *m *replicates were drawn from normal distributions for each network node. The means of these normal distributions were sampled from *N*(0.6, 0.01), if the node was expected to be perturbed according to the deterministic effects propagation scheme described above, and from *N*(0.95,0.01) otherwise. The variances of the normal distributions were drawn from Inv-*χ*^2^(4.4, 0.023). The choice of these parameters corresponds to those in Section "Methods" and is in agreement with our experimental data.

#### Network Reconstruction

**Figure 2:**
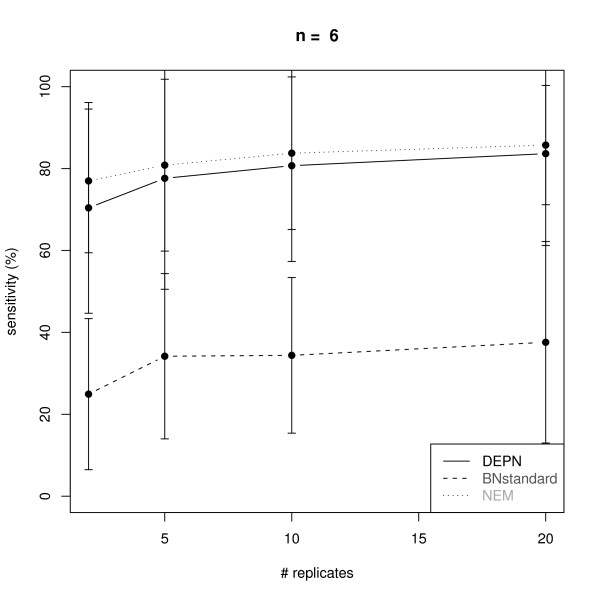
**Average sensitivity for the reconstruction of KEGG signaling networks with *n *= 6 nodes from artificial data via Deterministic Effects Propagation Networks (DEPNs), Bayesian Networks modeling directly dependencies between measurements (BNstandard), and Nested Effects Models (NEMs)**. Error bars indicate standard deviations.

**Figure 3:**
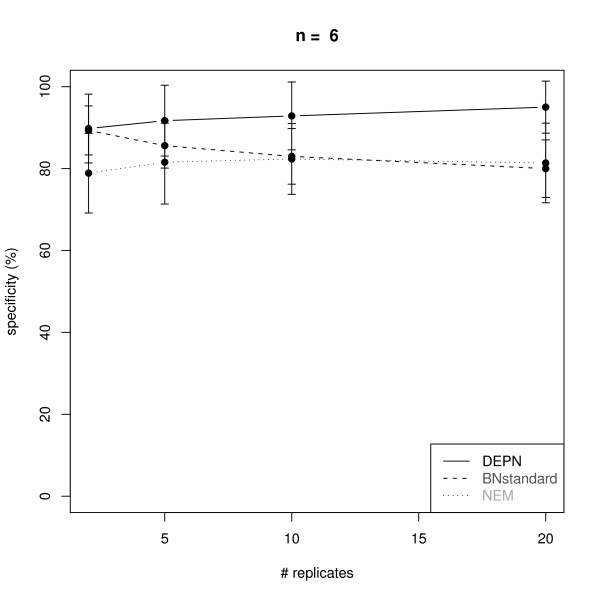
**Average specificity for the reconstruction of KEGG signaling networks with *n *= 6 nodes from artificial data via Deterministic Effects Propagation Networks (DEPNs), Bayesian Networks modeling directly dependencies between measurements (BNstandard), and Nested Effects Models (NEMs)**. Error bars indicate standard deviations.

**Figure 4:**
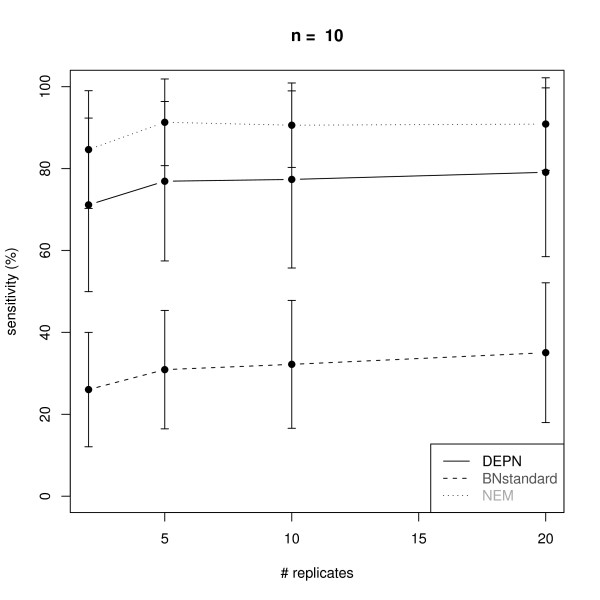
**Average sensitivity for the reconstruction of KEGG signaling networks with *n *= 10 nodes from artificial data via Deterministic Effects Propagation Networks (DEPNs), Bayesian Networks modeling directly dependencies between measurements (BNstandard), and Nested Effects Models (NEMs)**. Error bars indicate standard deviations.

**Figure 5:**
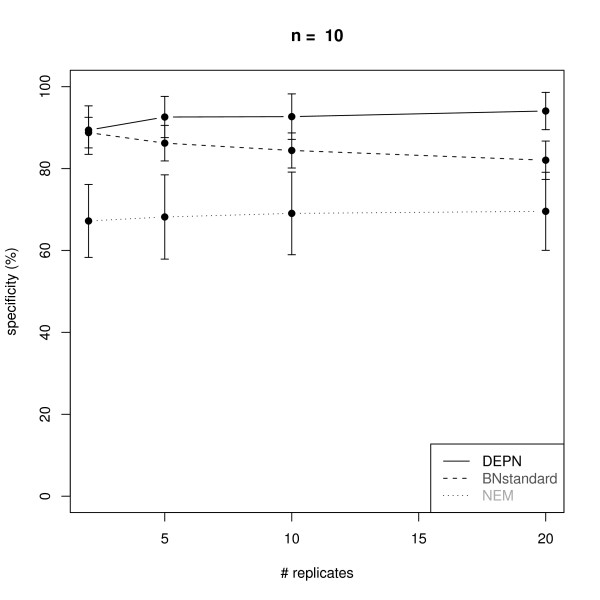
**Average specificity for the reconstruction of KEGG signaling networks with *n *= 10 nodes from artificial data via Deterministic Effects Propagation Networks (DEPNs), Bayesian Networks modeling directly dependencies between measurements (BNstandard), and Nested Effects Models (NEMs)**. Error bars indicate standard deviations.

Figures 2, 3, 4 and 5 visualize the performance of a network reconstruction with our approach for a varying number of replicates in terms of sensitivity (i.e. the rate of true positive edges) and specificity (i.e. rate of truly not inferred edges). For each number of replicates 100 networks with simulated perturbation data were generated as described before. As seen from the plots our method achieves a constantly high specificity of >90% with a sensitivity between 70% and 80%, i.e. the number of replicates has only a minor influence on the quality of the network reconstruction. The reason for this robust behavior is that due to the effects propagation scheme, we only operate in the space of transitively closed graphs. The restricted model class can be viewed as a kind of regularization [27] helping to identify a well performing network structure. Additionally, effects propagation increases the chance of getting enough data for both, the perturbed and the unperturbed case, for reliable parameter estimation.

We compared our DEPN approach to a standard implementation of Bayesian Networks, which directly models the dependency of measurements (called BNstandard in the following): For this purpose we discretized our simulated data (see "Data Simulation"). This was done by setting 0, if the data entry was closer to 0.6 (the expectation value of the perturbed data) than to 0.95 (the expectation value of the unperturbed data), and 1 otherwise. The conditional distribution at each network node was therefore a binomial with parameter equal to the perturbation probability of the node. We employed the R package *bnlearn *to learn the BN network structure via a hill climbing procedure initialized with an empty network. The Bayesian Dirichlet score was used to evaluate network structures. Please note, that BNstandard in contrast to DEPNs can only learn network structures without cycles. However, the vast majority of all original KEGG graphs were acyclic here (see "Graph Sampling").

Since our simulated data contains only single knock-outs, we also compared our DEPN approach to Nested Effects Models (NEMs): For this purpose we discretized our simulated data as before. The likelihood model introduced in [13] was employed with type I and type II error rates 0.06, which are expected by the known variances and means of the perturbed and unperturbed distributions. The same greedy hillclimber as for DEPNs was employed for network structure search.

**Figure 6:**
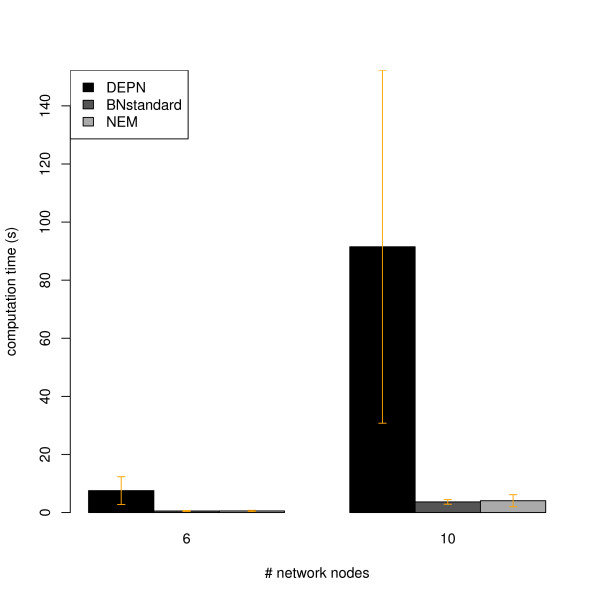
**Average computation times of DEPNs, BNstandard and NEMs on artifical data**. Error bars indicate standard deviations.

Figures 2, 3, 4 and 5 show a similar performance of BNstandard compared to DEPNs in terms of specificity, but a significantly worse sensitivity. The performance on acyclic graphs was essentially the same as on all graphs (including graphs with cycles). In terms of computation times BNstandard is clearly faster than the DEPN approach (Figure 6). Future optimizations of our DEPNs implementation may reduce this gap. Nevertheless, DEPNs are (up to now) only suitable for small network reconstructions. This is, however, in the light of the large experimental effort for multiple interventions and determination of their respective effects on network proteins not really a practical limitation.

In summary the comparison clearly demonstrates that a standard Bayesian Network approach, which models only conditional dependencies between measurements while ignoring the perturbation signal flow in the network, is not suitable for our kind of data.

Compared to NEMs, DEPNs show a significantly higher specificity and a slightly reduced sensitivity (Figures 2, 3, 4, 5). In terms of computation times, NEMs are clearly faster than DEPNs (Figure 6). This is expected, because NEMs can be formulated in a much more efficient way [13,20]. Since NEMs only employ single gene perturbations, they allow to perform deterministic effects propagation in an implicit rather than explicit way and do not have to estimate distribution parameters like DEPNs. Apart from that NEMs and DEPNs have a lot of similarities, but also major differences, which we highlight in Section "Related Work".

Altogether, both comparisons demonstrate that DEPNs seem to be well suited for the low dimensional knock-down effects data, which we are interested in here.

### Missing Values

**Figure 7:**
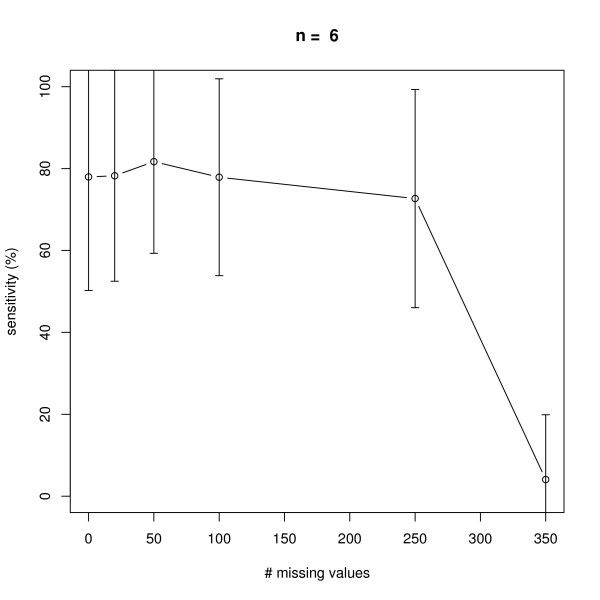
**Influence of the number of missing values on average sensitivity of network reconstruction via DEPNs for networks with *n *= 6 nodes**. Error bars indicate standard deviations.

**Figure 8:**
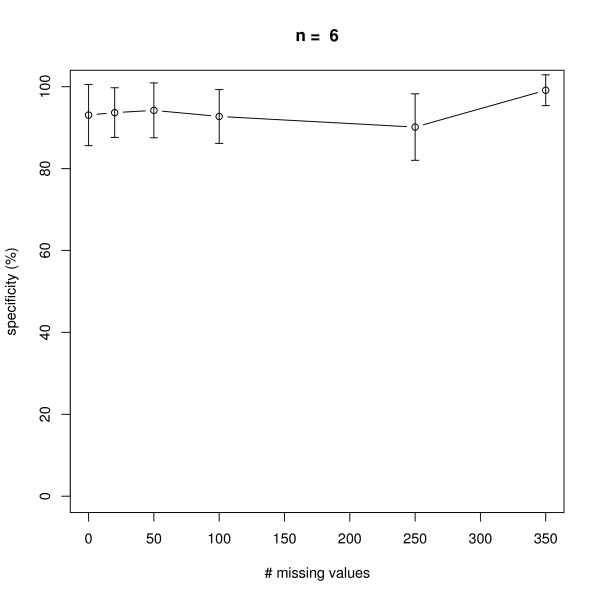
**Influence of the number of missing values on average specificity of network reconstruction via DEPNs for networks with *n *= 6 nodes**. Error bars indicate standard deviations.

**Figure 9:**
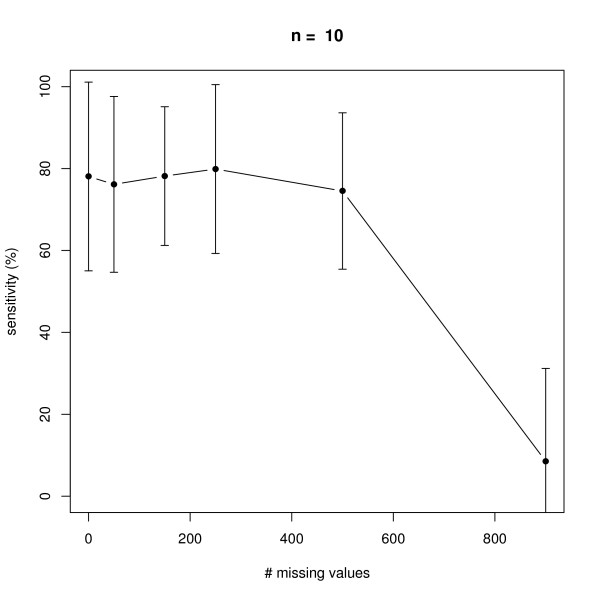
**Influence of the number of missing values on average sensitivity of network reconstruction via DEPNs for networks with *n *= 10 nodes**. Error bars indicate standard deviations.

**Figure 10:**
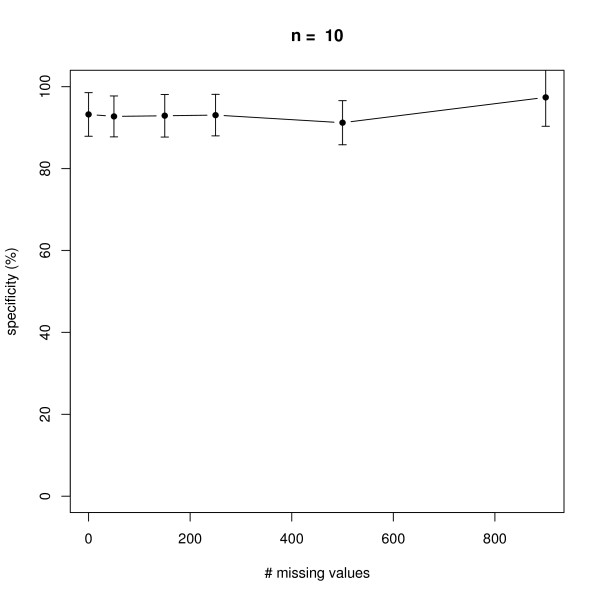
**Influence of the number of missing values on average specificity of network reconstruction via DEPNs for networks with *n *= 10 nodes**. Error bars indicate standard deviations.

We next investigated the effect of an increasing number of missing values in a dataset with 10 replicate measurements in networks with *n *= 6 and *n *= 10 nodes, i.e. 7 × 10 × 6 = 420 and 11 × 10 × 10 = 1100 data points in total (note that there is always one control experiment, where all nodes are unperturbed). The positions of missing values were chosen uniform randomly in the complete data matrix. As seen from Figures 7, 8, 9, 10 our method behaves very robust against replacing missing values with estimated posterior distribution mode values, even with 250 out of 420 *(n *= 6) and 500 out of 1100 *(n *= 10) missing values in total. Again, this robust behavior can be attributed to the same reasons as described before: a restricted model class together with the deterministic effects propagation scheme.

### Latent Nodes

**Figure 11:**
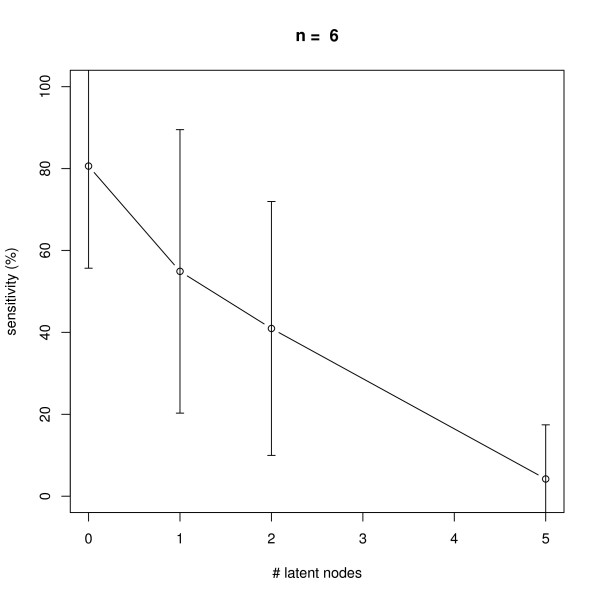
**Influence of the number of latent nodes on average sensitivity of network reconstruction via DEPNs for networks with *n *= 6 nodes**. Error bars indicate standard deviations.

**Figure 12:**
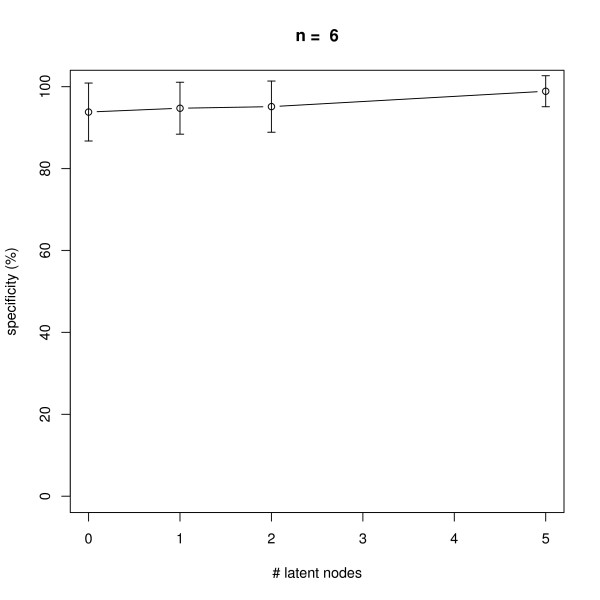
**Influence of the number of latent nodes on average specificity of network reconstruction via DEPNs for networks with *n *= 6 nodes**. Error bars indicate standard deviations.

**Figure 13:**
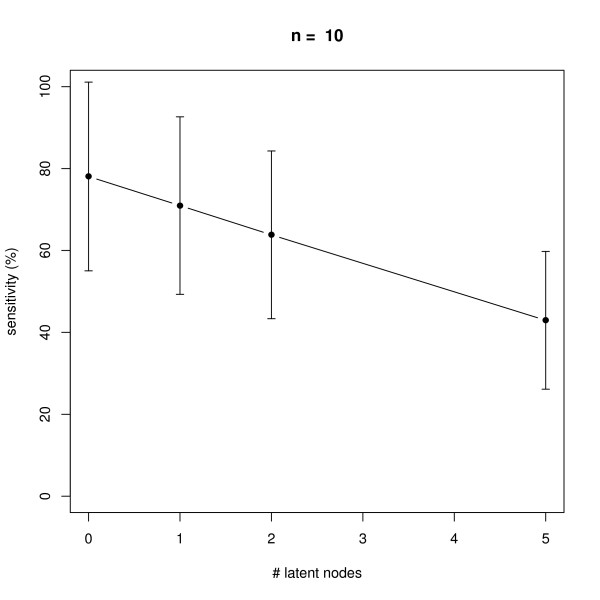
**Influence of the number of latent nodes on average sensitivity of network reconstruction via DEPNs for networks with *n *= 10 nodes**. Error bars indicate standard deviations.

**Figure 14:**
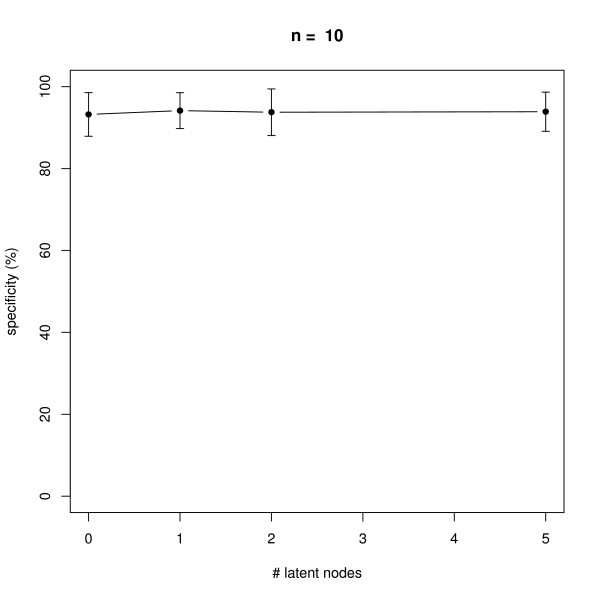
**Influence of the number of latent nodes on average specificity of network reconstruction via DEPNs for networks with *n *= 10 nodes**. Error bars indicate standard deviations.

We also monitored the effect of having latent nodes in the network. Again, data with 10 replicates was simulated in networks with *n *= 6 and *n *= 10 nodes. The results shown in Figures 11, 12, 13, 14 reveal a constantly high specificity of >90%, whereas the sensitivity drops with 1 latent node from ~80% to ~60% with *n *= 6 and ~80% to ~70% with *n *= 10, respectively. With 2 latent nodes we still achieve a sensitivity of ~40% with *n *= 6 and > 60% with *n *= 10. With 5 latent out of 6 nodes we have ~20% sensitivity, and with 5 latent out of 10 nodes we have ~40% sensitivity. We have to remind here that only outgoing edges from latent nodes can be estimated (see Section "Methods", Subsection "Latent Nodes" for details), hence the result shows an expected behavior. Note that on average with each latent node the number of unobservable edges should increase by roughly 1/6 = ~17% with *n *= 6 and by 10% with *n *= 10 (supposing the same average node degree). We believe that a positive point of our method is that still the specificity is rather high, meaning we do not get too many false positive edges.

### Reconstruction of the ERBB Receptor-Regulated G1/S Transition Network

#### RNAi and Reverse Phase Protein Arrays

**Figure 15:**
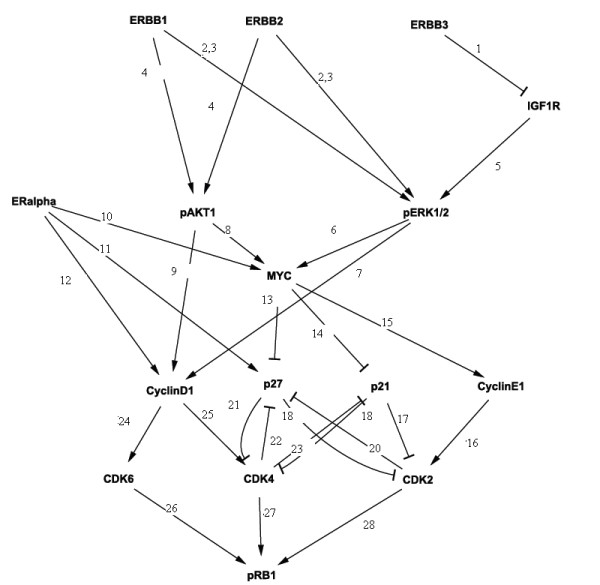
**Network of 16 proteins compiled from the literature**. Numbers point to corresponding references listed in a supplement to this paper [see Additional file 1].

Sixteen proteins playing a role in the G1/S transition of human cells were taken. Among these proteins there were the receptors *ERBB1, ERBB2 *and *ERBB3*, several signaling intermediates (e.g. *AKT1, MEK1) *and cell cycle proteins (e.g. *CDK4)*. Finally, we included the retinoblastoma protein *pRB1*, which regulates the G1/S transition. Interactions between these proteins known from the literature are shown in Figure 15 and Additional file 1.

Sixteen knock-downs (including 3 double knock-downs) with chemically synthesized siRNAs for proteins in our network and one experiment with MOCK transfected cells were conducted. After each siRNA transfection cells were stimulated with EGF for 12 hours. We measured the protein expression of signaling intermediates (in total 10 antibodies were available) for each knockdown sample using Reverse Phase Protein Arrays [23] before and after EGF stimulation with 4 technical and 3 biological replicates. These data were integrated using quantile normalization [28]. Further details of the experimental setup and a list of performed interventions can be found in the Material and Methods part of this paper. Additional information can be found in our earlier publication [25]. A heatmap of the data matrix is available in the supplement of this paper [see Additional file 2].

#### Network Reconstruction with DEPNs

**Figure 16:**
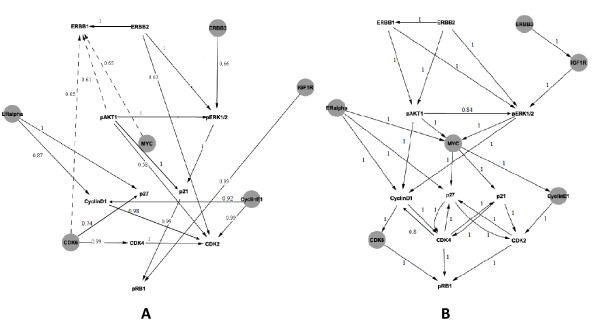
**A: Network learned by Deterministic Effects Propagation Networks purely from data**. Gray nodes indicate latent nodes without measurements. Dashed arrows were inferred from data, but cannot be explained by current literature knowledge. For the sake of better visualization we omit indirect edges between nodes, i.e. interactions, which can be explained by a cascade of others (transitive reduction). Numbers at edges indicate bootstrap probabilities. **B: Network learned by Deterministic Effects Propagation Networks from data when inference started with known interactions.** The dashed line indicates an interaction that cannot be explained by the literature network. Numbers at edges indicate bootstrap probabilities.

We used DEPNs to learn a network between the sixteen proteins purely from data. Since for 6 out of the 16 proteins in our network no measurements were available due to the lack of antibodies, they were marked as latent nodes (see Material and Methods and Figure 16). Furthermore, for *pERK1/2 *we had only 2 instead of 3 biological replicates for *Cyclin E1 *and *Cyclin D1 *knock-downs. Since our method in the current implementation requires the same amount of data for each experiment, measurements for the third biological replicate were treated as missing values and imputed during network learning using the EM algorithm approach described in the Materials and Methods part of this paper. Network structure learning was performed via greedy hill climbing.

In order to retrieve a measure of confidence for the individual edges learned by our method, we performed a nonparametric stratified bootstrap [29]. That means we sampled 100 times with replacement from each replicate group within each condition (stimulated/unstimulated). On each bootstrap sample (containing a subset of the complete dataset with possible duplicates) we estimated a network using DEPNs. After the bootstrap we counted the relative frequency of each edge in all 100 DEPN reconstructions. The relative frequency is an approximate probability for each edge (essentially the parameter of a binomial distribution). Only edges with a probability > 0.5 were considered as being reliable.

The result shown in Figure 16(A) reveals that our methods correctly recovered 20/29 interactions from the literature network with probability > 0.5, although we had 6 latent nodes. Here we also counted edges as correct, if they correspond to indirect interactions in the original network. This was valid, since DEPNs learn transitively closed graphs. The edge *ERBB2 *→ *ERBB1 *could be explained by the fact that *ERBB1 *and *ERBB2 *together form a heterodimer activating the *MAPK *and *PI3K *pathway [30,31]. Likewise, the edge *CDK6 *→ *CDK4 *was there, because both proteins form a complex [32]. The complex formation of *CDK4 *and *CDK6 *could also explain the edge *CDK6 *→ *p27 *[33]. It was found that *AKT1 *increases the expression of human *IGF1R *[34], which explained the edge *pAKT1 *→ *pERK1/2 *as an indirect activation of *pERK1/2 *by *AKT1 *through *IGF1R*. In conclusion there were only three edges, namely *CDK6 *→ *ERBB1, pAKT1 *→ *ERBB1 *and *MYC *→ *ERBB1 *for which we could not find any explanation in the literature.

We additionally performed a network inference starting from the literature network as an initial network for the greedy hill-climber (see Section "Methods", Subsection "Network Structure Learning"), i.e. we tried to learn additional edges from the data, which are missing in the literature network. This would be the intended and devised approach for using DEPNs practice, if possible. Like before, we applied the nonparametric stratified bootstrap to derive edge probabilities. As shown in Figure 16(B) the initialization with known edges from the literature made all three spurious edges dispensable to explain our data. Two edges were inferred in addition to the initial network: *pAKT1 *→ *pERK1/2 *and *CDK4: *→ *CyclinD1*. As mentioned before, *pAKT1 *→ *pERK1/2 *can be explained as an indirect activation of *pERK1/2 *by *AKT1 *through *IGF1R*. Furthermore, it has been observed that *CDK1 *and *CyclinD1 *can form a complex phosphorylating *pRB *[35], which explains *CDK4 *→ *CyclinD1*.

In conclusion of our experiments we have shown that DEPNs were able to reconstruct the ERBB-regulated G1/S transition network in a sufficient quality, even if there were many latent nodes.

The approach presented in this paper differs significantly from the one in our earlier publication on this data set [25], in which our aim was to explore biological implications of the literature network. This was done by comparing outcomes of knock-down simulations to our experimental data. Specifically, in our previous paper no likelihood model of the data was employed and therefore no network inference could be made. Both of these complementary modeling approaches, i.e. network reconstruction (here) and network based prediction of outcomes [25] demonstrate a good consistency of the literature network with the experimental data we have.

### Related Work

In this paper we introduced Deterministic Effects Propagation Networks as a special instance of Bayesian Networks for robustly estimating signaling networks from multiple and combinatorial intervention effects. DEPNs have certain similarities but also major differences to Nested Effects Models (NEMs) [13-21]. NEMs were especially designed to estimate signaling networks from high dimensional secondary intervention effects monitored on microarray data. The idea is to reverse engineer upstream signaling cascades from the nested structure of downstream effects, which is observed from several single gene interventions. The network is estimated between all perturbed genes.

Although our data in this paper looks relatively different at a first glance (i.e. relatively few proteins are monitored under single and combinatorial perturbations), there are also some similarities: If a certain protein *i *is perturbed, we expect to see a perturbation of all downstream proteins as well. That means the set of proteins being perturbed, if *i *is perturbed is a superset of the proteins being perturbed if any downstream protein of *i *is perturbed. In fact with our approach we implicitly learn this nested effects structure from data and encode it into a network. It should be mentioned that both, NEMs and DEPNs, can be interpreted within the Bayesian Networks framework in a similar way [18].

An additional feature of DEPNs compared to current NEM implementations is the possibility for latent network nodes. The advantage of this property is a more comprehensive view of the network of interest. However, it should be mentioned that it is only possible to estimate edges leading from latent nodes to measured nodes from data. Another major difference to NEMs is that DEPNs reconstruct the network between affected nodes from combinatorial perturbations of the same nodes. Therefore, DEPNs and NEMs may be seen as orthogonal approaches.

DEPNs are quite different from other Bayesian Network approaches for learning networks from intervention effects [6,7]: DEPNs do not assume a direct conditional dependence between measurement distributions. Instead DEPNs rely on a deterministic effects propagation scheme, which makes all measurement distributions statistically independent. Therefore, DEPNs can learn graphs structures containing loops, whereas usual Bayesian Networks are restricted to directed acyclic graphs. This is also an important difference to [6,7], which both employ ideal interventions [8]. These approaches assume to have few perturbations and a large amount of measurements (gene expression profiles) without interventions. In contrast, DEPNs make the opposite assumption: perturbations for almost all proteins in the network, but only few measurements (protein expressions) without interventions.

In contrast to mechanistic, quantitative approaches [12], DEPNs are probabilistic network models. DEPNs do not aim to model real protein concentration changes over time (usually involving a lot of a-priori knowledge on the system), but purely make probabilistic inference on the network structure given experimental data.

DEPNs differ significantly from reconstruction methods that overlay experimental data with known pathway information (e.g. [36]). In contrast DEPNs make a statistical inference of network structures from experimental measurements using a likelihood model.

## Conclusion

Studies of intervention effects play an increasing role in the understanding of complex biological networks. We introduced Deterministic Effects Propagation Networks (DEPNs) as a Bayesian Network computational approach to infer non-transcriptional signaling networks from multiple and combinatorial low dimensional intervention effects. In this paper these were quantitative proteomics data measured via Reverse Phase Protein Arrays. Our method hence aims at a system's understanding on the protein level. Nonetheless, DEPNs are in principle not limited to proteomics data and could be applied to other data showing similar characteristics.

Unlike Nested Effects Models [13-21], our method is especially designed for cases, where the number of perturbations exceeds the number of proteins in the network. Our method allows for the existence of latent nodes and of missing values. The reconstruction accuracy of DEPNs degrades with an increasing number of latent nodes within an expected range. This phenomenon is up to a certain degree unavoidable and common to all statistical approaches, since latent nodes imply a complete lack of data for one or several variables, which need to be deduced from available information. Apart from that our method was shown to be highly robust in our simulation studies. This specifically includes a very robust behavior against missing values.

We applied DEPNs to reconstruct the *ERBB *signaling network in trastuzumab resistant breast cancer cells from 16, partially combinatorial, siRNA interventions. The data were measured via Reverse Phase Protein Arrays. Although the data contained a high fraction of nodes without measurements, our network reconstruction was highly specific and showed only three spurious interactions. These could be made dispensable by starting the network reconstruction from current literature knowledge. Altogether our approach was able to reliable uncover a significant amount of the currently known *ERBB*-regalated human G1/S transition network, even with relatively little data.

In conclusion of our work we believe that DEPNs offer a robust and reliable approach to reverse engineer non-transcriptional signaling cascades from multiple low dimensional intervention effects. Compared to other Bayesian Networks, which model directly conditional dependencies between measurements, DEPNs showed a much higher network reconstruction accuracy in our simulation studies. At the same time DEPNs have a simpler inference mechanism, which allows for learning cyclic network structures in a straight forward way.

A limitation of DEPNs lies in the supposed deterministic way of effects propagation. Moreover, there is no inference made on the combinatorial functions carried out by the individual network nodes. Furthermore, DEPNs currently do not offer a model of the time dynamics (see [37] as an example for such an approach). While a model of the time behavior made no sense for our specific data, it is worthwhile to investigate dynamic DEPNs for different data types in the future.

Clearly, there are lots of other possibilities to improve the current method in the future. Besides inference on combinatorial functions of network nodes, this includes a more efficient way to deal with missing values and latent nodes, e.g. via structural EM [38], inference of up- and downregulated edges and improved network structure search strategies [39]. These issues should be investigated in the future.

The complete method as well as the data set is available as part of the R package "nem" [40] as a supplement to this paper [see Additional file 3].

## Methods

### siRNA Transfections and EGF Stimulation

HCC1954 cells (CRL-2338, from ATCC) were cultured in RPMI 1640 Modified Medium which is supplemented with 50 U/mL penicillin, 50 g/mL streptomycin sulphate, 1% non-essential amino acids and 10% fetal bovine serum (all media and supplements from Gibco BRL). The cells were seeded at a number of 7 × 10^5 ^for 24 hours before transfection. Cells were transfected with 20 nM siRNA pool for each gene (except ESR1, 50 nM) and 25 *μ*L Lipofectamine 2000 transfection reagent (Invitrogen, Carlsbad, CA). For further experimental details, validation of the used cell line, siRNA and prime sequences, see [25]. After transfection, cells were synchronized using Dif-3 (30 *μ*M, Sigma) for 22 hours in medium containing 10% FBS. Cells were further starved in 0% FBS medium for 2 hours. After 24 hours of starvation, cells were stimulated with EGF (25 ng/mL) for 6, 12, 18 and 24 hours.

In total 16 gene knock-downs were performed: *AKT1, MEK1, CDK2, CDK4, p21, p27, Cyclin D1, ERBB2 + ERBB3, ERBB1 + ERBB3, ERBB1, ERBB1 + ERBB2, IGF1R, CDK6, ER-a, c-MYC, Cyclin E1*.

### Cell lysis and Reverse Phase Protein Arrays

The cells were lysed on ice by scraping the cells in M-PER lysis buffer (Pierce, Rockford, IL) containing protease inhibitor Complete Mini (Roche, Basel), anti-phosphatase PhosSTOP (Roche, Basel), 10 mM NaF and 1 mM Na4VO3. Protein concentrations were determined with a BCA Protein Assay Reagent Kit (Pierce, Rockford, IL). Lysates were mixed 1:2 with 2× Protein Arraying Buffer (Whatman, Brentfort, UK) to obtain a final protein concentration of 1.5 *μ*g/*μ*L. Briefly, these lysates were printed onto nitrocellulose coated ONCYTE-slides (Grace Bio Labs, Bend, USA) using a non-contact piezo spotter, sciFlexxarrayer S5 (Scienion, Berlin, Germany). After primary and near-infrared (NIR)-dye labeled secondary antibodies applied, spots were analysed using an Odyssey scanner (LI-COR, Lincoln, USA) and signal intensities were quantified using Odyssey 2.0 software. Further information and an antibody list can be found in [25]. Since no antibody against *MEK1 *was available, we measured protein expression of *pERK1/2*, which is downstream of *MEK1*.

### Network Inference with Deterministic Effects Propagation Networks

#### Likelihood Model

Each protein corresponds to one node in our network graph Φ. We suppose measurements  for the *i*th protein at time point (or experimental condition - e.g. stimulated/unstimulated) *t *under perturbations *p (p *are the indices of perturbed nodes) to be drawn from a Gaussian distribution with unknown mean and variance:

(1)

where *pa(i) *denotes the set of parents of node *i*. The formula means that perturbations are propagated in a deterministic fashion from parents to direct children, i.e. a node is considered as perturbed whenever either itself or any of its parents was perturbed. If we have an edge *a *→ *b *and *a *is perturbed we expect to observe an effect for measurements of *a *and *b*. These measurements for each time point are drawn from two normal distributions, one for the perturbed and one for the unperturbed case. Whether a given data point is drawn from the one or the other normal distributions only depends on the perturbation state of the network node and therefore on the network structure.

Eq. 1 assumes all parents to cause the same directional effect (activation or inhibition) on children. However, this simplification can easily be overcome by introducing two distributions for the perturbed case: one, if an activation is expected and one, if an inhibition is expected. In order to simplify the explanation of our approach we here only refer to the binary perturbed/non-perturbed case.

Unlike other Bayesian Network approaches [41,42] we do not assume a direct dependency of  from measurements of other proteins. Instead,  only depends only on the perturbation state of node *i*, which itself depends on the perturbation state of its parent nodes via the network structure Φ in a deterministic way. We believe that this assumption does not only simplify our calculations, but also leads to a better robustness against noise, e.g. due to differences in protein antibody sensitivities. Our modeling approach allows us to directly make use of the supposed flow of perturbation signals for network graph likelihood calculation (see below). Further, our approach allows for arbitrary directed network graph structures, i.e. also cyclic networks, which are often observed in biological signaling pathways.

The data  implicitly may also depend on the measurements at the previous time point *t *- 1. However, we do not model this time dependency here, since this would require long time course data.

If the network structure Φ is known, the parameters (, ) and (, ) can be estimated in two ways. For the sake of brevity in the following we only talk about (, ), but of course the same also holds for (, ):

1. Maximum likelihood estimate:  =  and  = , where  and  are the empirical (unbiased) mean and standard deviation, respectively.

2. Bayesian estimate: We suppose | ~*N *(*μ*_0_, /*λ*_0_)and  ~Inv-*χ*^2^(*α*_0_, *β*_0_), where *μ*_0_, *λ*_0_, *α*_0 _and *β*_0 _should be chosen in dependency of the perturbation state. According to the expectations for our data we set *μ*_0 _= 0.95, *λ*_0 _= 4, *α*_0 _= 4.4, *β*_0 _= 0.023 for the unperturbed and *μ*_*o *_= 0.6, *λ*_0 _= 4, *α*_0 _= 4.4, *β*_0 _= 0.023 for the perturbed state. This corresponds to an expected *σ*_*i *_of 0.2 with a standard deviation of 0.1. The marginal posterior distributions for  and  can be calculated in analytical form [43]:

(2)

(3)

where (*μ*_*n*_, *β*_*n*_/*λ*_*n*_) denotes the Student-*t *distribution with *α*_*n *_degrees of freedom, location *μ*_*n *_and scale *β*_*n*_/*λ*_*n*_. Inv-*χ*^2 ^denotes the scaled inverse *χ*^2 ^distribution, and parameters *μ*_*n*_, *λ*_*n*_, *α*_*n *_and *β*_*n *_are given as:

(4)

(5)

(6)

(7)

where *n*_*i *_is the number of observations used to compute the conditional density for . The posterior modes of | and | are *μ*_*n *_and .

Let us now assume we have a data set *D *= {} of measurements of all proteins at all time points under varying perturbations. Let *θ *denote the vector of all estimated posterior mode parameters. Let  denote the the set of all perturbations. The likelihood of a network hypothesis is then calculated as:

(8)

where *T *is the number of time points, *n *the number of nodes and *r *the number of replicate measurements.

### Missing Data Imputation

Missing data points are an often observed problem in real life data. We use an EM algorithm [44] to address this issue. The first iteration of the EM-algorithm works as follows: In the M-step we infer the parameters for a given network structure as described before. In the E-step we calculate the likelihood of the data under the given network structure when filling in posterior mode values for the missing data points. The posterior modes of | and | are *μ*_*n *_and  (see above). In the next iteration of the EM algorithm in the M-step we infer parameters on the data with missing values being replaced. Based on these parameters we get new posterior mode parameters, which we then fill in for the values marked as missing before. The whole procedure is repeated until convergence.

#### Latent Network Nodes

Our method also allows for a scenario, in which we have performed knockdowns on proteins without available antibodies and thus without direct available measurements. Nevertheless, these latent network nodes can have an influence on the other nodes, and this influence can be quantified indirectly by perturbing them and measuring the downstream influence on the other network members. This way it is possible to estimate outgoing edges from latent nodes: Please note that in our model perturbations are propagated from parents to children, i.e. whenever there is an edge between two nodes *a *→ *b *and *a *is perturbed, we expect to see an effect in all measurements of *b*. Assume there is no data given for *a*. Nevertheless, the edge *a *→ *b *can be necessary to explain the data for *b*. Hence, a network hypothesis including this edge will receive a higher likelihood than another one without this edge.

Practically, we do not need to do anything different in the case we have latent nodes than in the case we have full observations: Given a network hypothesis Φ we propagate expected downstream perturbation effects and calculate the likelihood of observing our data according to the perturbation state of all network nodes.

#### Network Structure Learning

Network structure learning was performed in a greedy hill climbing fashion: Beginning from an initial network (which is, if not mentioned otherwise, the empty network) we successively added that edge, which increased the likelihood function Eq. (8) most. Afterwards the transitive closure of the new graph was calculated, which was necessary in order to perform learning within the space of transitively closed graphs. This process was continued until no improvement could be gained any more. Please note that in the space of transitively closed graphs there is no clear way of performing edge deletions and reversals. Hence, we restricted ourselves to edge insertions.

## Authors' contributions

DEPNs were invented by HF. CB gave support with data analysis and helpful discussions. ÖS conducted the experiments. DA and TB initiated and guided the project. All authors have read and approved the manuscript.

## Supplementary Material

Additional file 1 **References for Network**. Table with literature references for interactions between network proteins. Click here for file

Additional file 2 **Matrix View**. Matrix view of the RPPA data. Click here for file

Additional file 3 **DEPN implementation**. NEM software package containing DEPN implementation. Click here for file
