# Supplementary material for: Deterministic Effects Propagation Networks for reconstructing protein signaling networks from multiple interventions
Source: BMC Bioinformatics. 2009 Oct 8;10:322. doi: 10.1186/1471-2105-10-322 (PMC2770070; doi:10.1186/1471-2105-10-322)
Supplement: Additional file 1 — References for Network. Table with literature references for interactions between network proteins. [file 1471-2105-10-322-S1.DOC]

| 1 | ERBB2/ERBB3 protein decreases expression of IGF1R mRNA | Alaou-Jamali MA et al., 2003 (Cancer Research) |
| --- | --- | --- |
| 2 | ERBB1/ERBB2 heterodimers are potent activating the Ras/mitogen-activated protein kinase pathway | Zhan L et al., 2006 (Cancer Research) |
| 3 | ERBB1/ERBB2 heterodimers can activate MAPK pathway by docking Grb2/Ras proteins | Alroy I and Yarden Y., 1997 (FEBS Lett) |
| 4 | ERBB1/ERBB2 heterodimers are potent in activating the phosphoinositide 3’-kinase (PI3K) | Zhan L et al., 2006 (Cancer Research) |
| 5 | IGF1R activates docking substrate Shc which activaes SOS, Ras and downstream Raf/MEK/ERK cascade | Shelton JG et al., 2004 (Cell Cycle) |
| 6 | Ras/Raf/MEK/MAPK pathway regulates C-MYC expression | Liu MM et al., 2002 (J Biol Chem) |
| 7 | Sustained ERK activity increases Cyclin D1 mRNA and protein level / Raf/MEK/ERK cascade increases transcription of Cyclin D1 | Weber JD et al., 1997 (BiochemJ) / Coleman et al., 2004 |
| 8 | PI3K/AKT pathway stimulates translation of C-MYC mRNA and stabilization of the protein | Sears R et al., 2000 (Genes Dev.) |
| 9 | Inhibition of PI3K by LY294002 decreases the half-life of the 4.5 kb Cyclin D1 mRNA / AKT stabilizes Cyclin D | Dufourny B et al., 2000 (J. Endocrinology) |
| 10 | Induction of C-MYC through ER-alpha leads to activation of Cyclin E/CDK2 complex via reduction of p21 | Sutherland RL et al., 1998 (J Mammary Gland Biol Neoplasia) |
| 11 | Adenoviral expression of ER-alpha in ER-negative breast cancer cells leads to increase in protein levels p27 cyclin-dependent kinase inhibitor | Licznar A et al., 2003 (FEBS Lett) |
| 12 | ER activation at the Cyclin D1 promotor is mediated by both the cylic AMP-response element and the activating protein 1-site | Lie MM et al., 2002 (J Biol Chem) |
| 13 | MYC acts as an upstream regulator of CDKs and functionally antagonizes the action of p27 | Maddika S et al., 2007 (Drug Resistance Updates) |
| 14 | Interaction of MYC-MAX heterodimer with MIZ-1 and/or SP-1 inactivates p21 | Pelengaris et al., 2002 (Nature Reviews Cancer) |
| 15 | Induction of C-MYC leads to activation of Cyclin E/CDK2 complex via reduction of p21 | Prall OW et al., 1998 (MCB) |
| 16 | Cyclin E binds to and activates CDK2 to regulate G1/S transition | Ohtsubo M et al., 1998 (Mol Cell Biol) |
| 17 | P21 inhibits the activity of CDK2 | Harper JW et al., 1995 (MCB) |
| 18 | P21 inhibits the activity of CDK4 | Harper JW et al., 1995 (MCB) |
| 19 | P27 prevents the activation of CDK2 | Cariou S et al., 2000 (PNAS) |
| 20 | The Cyclin E-CDK2 complex positively regulates its activity by phosphorylating p27, which is then targeted for degradation | Sherr CJ and Roberts JM, 2004 (Gene Dev.) / Müller D et al., 1997 (Oncogene) |
| 21 | P27 inhibits the activity of CDK4 | Cariou S et al., 2000 (PNAS) |
| 22 | The growth factor activation of Cyclin D/CDK4 complex sequesters unbound p27 and inhibits its inhibitory effect of Cyclin E/CDK2 complex | Grillo M et al., 2006 (Breast Cancer Research and Treatment) |
| 23 | The growth factor activation of Cyclin D/CDK4 complex sequesters unbound p21 and inhibits its inhibitory effect of Cyclin E/CDK2 complex | Grillo M et al., 2006 (Breast Cancer Research and Treatment) |
| 24 | Cyclin D1 binds to CDK6 to activate its kinase activity | Vermeulen K et al., 2003 (Cell Prolif) |
| 25 | Cyclin D1 binds to CDK4 to activate its kinase activity | Morgan DO et al., 1997 (Ann Rev Cell Dev Biol) |
| 26 | Activated Cyclin D/CDK4 or CDK6 complex phosphorylates pRb, releasing it from its growth suppressive functions | Yu B et al., 2000 (Mol Cell Biol Res Com) |
| 27 | Cyclin D/CDK4 complex phosphorylates pRB protein at Ser807/811 | Zarkowska T et al., 1997 (J Biol Chem) |
| 28 | The activated Cyclin E/CDK2 complex also contributes to the phosphorylation of pRB late in G1 | Hatakeyarna M, 1994 (Genes Dev) / Hinds P et al., 1998 (PNAS) |

**Table 1:** References for the edges in our literature network
