# Supplementary material for: Deterministic Effects Propagation Networks for reconstructing protein signaling networks from multiple interventions
Source: BMC Bioinformatics. 2009 Oct 8;10:322. doi: 10.1186/1471-2105-10-322 (PMC2770070; doi:10.1186/1471-2105-10-322)
Supplement: Additional file 2 — Matrix View. Matrix view of the RPPA data. [file 1471-2105-10-322-S2.DOC]

# No EGF EGF stimulated

**Figure:** Matrix view of the RPPA data. Columns contain interventions (MOCK is the control) and rows measured responses. Each column tick marks 12 replicate measurements. First and second half of the matrix corresponds to the unstimulated and EGF stimulated condition. The matrix visualizes fold changes to the unstimulated control (i.e. a fold change of 0.5 means a 50% reduction to the control). Higher values are marked darker, lower values lighter.
